# Hypothesis on ontogenesis and pathophysiology of Alzheimer’s disease

**DOI:** 10.31744/einstein_journal/2022RW0170

**Published:** 2022-11-09

**Authors:** Eduarda Dal Pisol Schwab, Ruliam Queiroz, Anne Karine Bosetto Fiebrantz, Murilo Bastos, Juliana Sartori Bonini, Weber Cláudio Francisco Nunes da Silva

**Affiliations:** 1 Universidade Estadual do Centro-Oeste Guarapuava PR Brazil Universidade Estadual do Centro-Oeste, Guarapuava, PR, Brazil.

**Keywords:** Alzheimer disease, Oxidative stress, Amyloid beta-peptides, Tau proteins, Apolipoprotein E4, Glycogen synthase kinase 3, Cyclic AMP response element-binding protein, *Diabetes mellitus* type 3, Endocannabinoids, Insulin resistance

## Abstract

Alzheimer’s disease is a neurodegenerative condition that causes changes in memory and cognition, in addition to behavioral disorders, and most commonly affects the elderly. Several studies in the literature have presented therapeutic measures in an attempt to interfere with the pathogenic mechanisms of the disease and to mitigate its clinical manifestations. Some factors, such as excitotoxicity, cholinergic dysfunctions, oxidative stress, tau protein hyperphosphorylation, changes in amyloid-beta peptide metabolism, herpes viruses, apolipoprotein E, glycogen synthase kinase 3, insulin resistance, and the endocannabinoid system seem to be related to pathophysiology of Alzheimer’s disease. Given this, a literature review was carried out to address the molecular mechanisms associated with the pathophysiological hypotheses previously mentioned, aiming to better understanding their underlying causes and contributing to possible pharmacological strategies about treatment of the disease.

## INTRODUCTION

Alzheimer’s disease (AD) is a neurodegenerative condition that presents itself through a decline in cognitive ability, progressive memory loss, and behavioral changes.^(1,2)^ It was first described in 1907, by Alois Alzheimer, a Bavarian psychiatrist, based on clinical and histopathological features observed in the brain of a patient with cognitive and memory difficulties at the age of 51 years.^(3,4)^ Currently, AD is the most common type of dementia, affecting mainly the elderly population over 65 years and impairing aspects related to attention, language, learning, memory, and judgment.^(5,6)^ It dramatically affects the patient’s individuality, since it gradually makes them dependent on help of family members and caregivers to perform daily activities, from the simplest to the most complex tasks.^(7)^

Worldwide, approximately 36.5 million people have dementia, mainly related to AD.^(8,9)^ The global prevalence of dementia has been estimated at 3.9% in people aged over 60 years, with a regional prevalence of 1.6% in Africa, 4.0% in China and Western Pacific regions, 4.6% in Latin America, 5.4% in Western Europe and 6.4% in North America.^(10)^ In Brazil, the age-standardized prevalence rate of AD is 1037/100,000.^(11)^ In 2013, AD ranked sixth as cause of death in the United States, accounting to 84,767 patients affected by worsening of the disease. Moreover, it is estimated that by 2050, only in the United States, the population affected by some form of dementia will be 13.8 million.^(8,12)^ Life expectancy in the world is increasing, and, consequently, the number of neurodegenerative conditions.^(13)^ Each year, approximately 5 million new AD cases are reported worldwide.^(14)^ Thus, governmental and family costs associated with AD are increasingly high since patients require drug therapy and a multi-professional approach to prevent the progression of the disease and ameliorate its effects on the central nervous system.^(15,16)^

Several factors have been reported in the literature indicating possible mechanisms for AD, including oxidative stress,^(17)^ abnormal calcium influx into the intracellular milieu,^(18)^ cholinergic dysfunctions,^(19)^ herpes simplex virus infections,^(20,21)^ beta-amyloid deposition^(22)^ and tau protein hyperphosphorylation.^(23)^ Adjacent to the intracellular findings of neurofibrillary tangles and extracellular senile plaques in the AD-affected brain, microscopic findings indicate beta-amyloid peptide and tau protein appear to play an important role in pathophysiology of Alzheimer,^(24–26)^ although it is not yet possible to state a direct relation between these factors and the disease.^(27)^ There is no curative treatment for AD, only symptomatic measures, and the disease has a remarkable impact on the healthcare system and families´ structure and dynamics; therefore, the number of studies aimed to clarify and deepen understanding of molecular aspects of the origin of the disease, as well as to develop a possible effective therapeutic agent against the progressive neurodegeneration caused by AD has increased.^(28)^ Based on this, this article aims to make a review of state-of-the-art addressing the pathophysiological mechanisms of Alzheimer’s disease described in the literature, in increasing order of therapeutic potential to be explored, to contribute to development of possible pharmacological measures to reduce the clinical symptoms of the disease.

This integrative literature review was carried out by searching in PubMed, EMBASE, LILACS,, and SciELO databases. The articles included in this study were found using the keywords “Alzheimer disease”, “cholinergic”, “beta-amyloid”, “tau protein”, “excitotoxic”, “oxidative stress”, “APOE4”, GSK3, “CREB”, “type 3 diabetes”, “endocannabinoid system” and “hypothesis” combined with the Boolean OR operator, independently or together. The following were included in the results of this review: articles published in Portuguese and English, with no defined time interval, and full text available for reading. The exclusion criteria were articles that did not cover the topic, case reports, and letters to the editor.

### Cholinergic hypothesis

Based on the reduction of cholinergic function, this hypothesis arose in the 1970´s, after *post-mortem* observation of human brain tissues of people with AD.^(29)^ Some studies have shown that AD patients reduce choline acetyltransferase rates, especially in cortical and hippocampal regions, which are punctual neurodegeneration sites of the disease.^(30)^ A study prior to that by Bowen et al., also pointed out reduced levels of the enzyme in individuals with cognitive dementia.^(29)^

Choline acetyltransferase is the enzyme responsible for choline acetylation to form the neurotransmitter acetylcholine. After its generation, the transmitter is transported by monoamine transporters and stored in vesicles, which undergo exocytosis due to calcium influx when the membrane is depolarized. Acetylcholinesterase is responsible for degrading the neurotransmitter present in the synaptic cleft, so that it is recycled and reused by the presynaptic terminal.^(31)^

It is reported in the literature that the loss of cognitive function in AD can still be caused and aggravated by changes in choline transport, neurotransmitter release, and expression of nicotinic and muscarinic receptors.^(32)^ M2 receptors, more numerous in presynaptic terminals, appear to be more affected than M1 receptors in presynaptic and postsynaptic terminals. The α7 postsynaptic nicotinic receptors influence the formation of memories and learning process, and their loss can contribute to dementia in AD.^(30,33)^ In addition, some *in vitro* studies show an inverse relation between cholinergic transmission in the hippocampus and cortex and the levels of beta-amyloid protein, given that it generates neurotoxicity with consequent impairment of the synthesis and release of acetylcholine.^(34,35)^

The cholinergic hypothesis is intrinsically linked to the early stages of AD.^(30)^ Research indicates that the initial asymptomatic or prodromal stage of AD is mainly related to presynaptic dysfunction of cholinergic neurons in the nucleus basalis of Meynert, which receives limbic afferents and projects fibers onto other cortical regions of the brain.^(19,36)^ This cholinergic innervation significantly contributes to the activation of the extrathalamic ascending reticular system, which demonstrates the importance of acetylcholine for the states of wakefulness, attention, and consequent formation of memories. Other recent studies have also linked the nucleus basalis of Meynert with cholinergic loss in AD.^(37–39)^

Considering the cholinergic neuron dysfunction in AD patients, acetylcholinesterase has been seen as a possible therapeutic target to increase concentrations of the neurotransmitter in the synaptic cleft, leading to its greater availability to act in nicotinic and muscarinic receptors. The most frequent globular forms of acetylcholinesterase in the brain as a whole are G1 and G4, but there is a reduction in the levels of the G4 form in AD, suggesting the G1 form of acetylcholinesterase is the most viable therapeutic target.^(40)^ However, no studies have proved these anticholinesterases promote regression of the disease; they only bring temporary relief of symptoms, especially in the early stages.^(41)^

In addition, it is essential to highlight that the loss of cholinergic function and dementia does not necessarily indicate a causal relation, and possible connections between these factors should be better studied.^(36)^

### Beta-amyloid hypothesis

The beta-amyloid peptide, composed of 40 to 42 amino acids, is derived from a transmembrane protein called amyloid precursor protein (APP), which has a gene located on the long arm of chromosome 21 and is intrinsically related to memory, neuronal plasticity, and adhesion between neurons and extracellular matrix.^(42)^ It has two domains: C-terminal (internal) and N-terminal (external).

Amyloid precursor protein (APP) can be metabolized in two ways: non-amyloidogenic and amyloidogenic. In the first, APP is cleaved by α-secretase enzymes, which generate neuroprotective products, such as α-APP and C-83, and, in turn, generate P-83 and APP intracellular domain, and γ-secretase, which results in beta-amyloid accumulation.^(30)^ On the other hand, the amyloidogenic pathway has the participation of β-secretase enzymes - mainly the BACE1 form, abundant in the brain - and γ-secretase, which makes the proteolysis of APP and generate the β-sAPP fragment, a contributing factor to the increased amount of beta-amyloid peptide in the brain, and C99-AP. The β-sAPP fragment is cleaved by γ-secretase and produces an beta-amyloid 42 isoform, which accumulates and contributes to neuroinflammation and microglia activation.^(30,43)^ Dysfunctions caused by mutations in the APP gene significantly affect the amyloidogenic pathway and cause an uncontrolled increase in peptides in neuronal tissue, so that it deposits and forms insoluble senile plaques, especially in the early stages of AD.^(44)^ In addition, mutations in this transmembrane protein can interfere with generating its isoforms, which occurs by alternative splicing.^(45)^

The clearance of beta-amyloid is impaired by several factors, with emphasis on the genetic predisposition of AD patients, who may eventually carry the apolipoprotein E4 (ApoE4), PICALM, and APOJ genes, in addition to presenilin polymorphisms (PSEN1 and PSEN2), which are subunits of γ-secretase.^(22,46,47)^ Despite their neuroinflammatory actions, there is not enough scientific evidence to demonstrate a direct causal relation between the presence of beta-amyloid oligomers and AD, although these can trigger neurodegenerative and toxic conditions characteristic of the disease. It is reported in the literature that beta-amyloid peptide catalyzes enzymes that contribute to the hyperphosphorylation of the tau protein, such as glycogen synthase kinase 3 (GSK3).^(48)^ The use of secretase inhibitors has been a widely studied strategy, as well as peptide anti-aggregation and antibodies to beta-amyloid.^(49,50)^

### Tau protein hypothesis

Tau protein is a microtubule-associated protein consisting of 16 exons, which play an essential role in maintaining stability of neurons in the central and peripheral nervous system by means of polymerization of tubulin. This protein is very susceptible to interaction with other proteins, in addition to being subject to several modifications after the translation process, such as acetylation and glycosylation.^(51)^ Its gene is located on chromosome 17q21.31 and has six isoforms produced by alternative splicing,^(52)^ with three major domains that differ in number of amino acids.^(53)^

Under physiological conditions, the tau protein exists in the phosphorylated form in the axon.^(30)^ The addition of phosphate to the molecule occurs through the esterification of tyrosine, serine, and threonine residues. Among the main enzymes involved in this process are protein kinase A (PKA), c-Jun amino-terminal kinase (JNK), tau protein kinase I (TPKI), GSK3, dual-specificity tyrosine phosphorylation-regulated kinase 1A (DYRK1A), and AMP-activated protein kinase (AMPK).^(52)^ The presence of inflammatory mediators, such as TNF-alpha, F-KB), and interleukins, contributes to the activity of the mitogen-activated protein kinase (MAPK) and CDK-5). *In vitro* studies have shown that casein kinase can also phosphorylate tau protein.^(54,55)^

There is evidence of an accumulation of tau protein in central nervous tissues affected by AD.^(56,57)^ In addition, it has been reported that a hyperphosphorylation process of this protein occurs through the kinase overload in patients with cognitive decline characteristic of AD. Hence, the protein dissociates from the microtubules and initiates a process of cell injury that invariably leads to cell death.^(58)^ The aggregation of phosphorylated tau proteins interferes in neurofibrillary tangles, essential mediators of neurodegeneration, and in neuronal dysfunction in the brain affected by AD.^(59)^

### Excitotoxic hypothesis

Glutamate is the excitatory neurotransmitter in the brain, responsible for synaptic plasticity and processes related to memory and learning.^(60)^ It can be produced over several signaling pathways within the central nervous system,^(61)^ and is prevalent in some regions, such as hippocampus and cerebral cortex.^(30)^ This neurotransmitter has two types of receptors: ionotropic and metabotropic. Ionotropic glutamate receptors (iGluRs) are called N-methyl-D-aspartate receptors (NMDA) and play an essential role in development of cognitive disorders, especially AD, since they participate in the processes of formation and modulation of memory.^(61,62)^

In general, when the membrane is polarized with a potential of -70mV, the voltage dependent Ca^2+^ channels of NMDA receptors are blocked by Mg^2+^. After depolarization of the membrane, the block by magnesium ions is removed, and there is an influx of calcium into the cell.^(63)^ In AD, there is hyperexcitability of the channels of these receptors, caused by deficient recycling of glutamate released in the synaptic cleft. These mechanisms cause the neurotransmitter to keep acting on the receptors chronically and moderately, activating them and triggering an influx of excessive Ca^2+^ ions into the neuronal cell. The increased concentration of intracellular calcium damages the cells causing degeneration and cell death.^(61,64)^ Some organelles, such as mitochondria and endoplasmic reticulum, are extremely sensitive to intracellular changes in calcium.

Some factors seem to contribute to increased excitotoxicity caused by glutamate, such as suppression of genes involved in the processes of cell death, activation of calpain (a calcium-dependent protein related to apoptosis), production of inflammatory mediators and free radicals related to oxidative stress through activated phospholipase A2, in addition to decreased cell energy metabolism with a consequent reduction in intracellular energy levels and ATP-dependent membrane transport.^(30,65)^

### Oxidative stress hypothesis

The presence of biomarkers for oxidative stress, such as 3-nitrotyrosine, 8-hydroxyguanosine, and 8-hydroxydeoxyguanosine, has been reported in the blood of AD patients and animal models.^(66)^ The participation of this phenomenon in AD-related neurodegeneration is very evident in the literature, since cells are very vulnerable to changes in tissue homeostasis, especially those caused by the increase in reactive oxygen species,^(30)^ which exceed the barrier established by the cellular antioxidant defense system. Given its high level of oxygen consumption, neuronal tissue tends to be significantly affected by free radicals generated by oxidative stress.^(17)^

Oxidative stress, in general, can be defined as an imbalance between oxidizing and antioxidant factors, which generates inflammatory products and several other components that functionally interfere in cells.^(67)^ The mechanisms that initiate this imbalance have not yet been fully elucidated,^(66)^ although some studies have suggested that factors, such as DNA oxidation, dysfunctions in the energy metabolism of mitochondria, aggregation of beta-amyloid peptides, and lipid peroxidation, can trigger and aggravate oxidative lesions in the brain, especially in some regions, such as cortex and hippocampus.^(30)^

The accumulation of beta-amyloid peptides has been closely related to mitochondrial changes that generate free radicals, mainly because it interferes with the electron transport chain and causes irreversible neurodegenerative lesions. In addition, the formation of reactive oxygen and nitrogen species due to the deficiency and abnormal function of cytosolic and membrane enzymes, significantly contributes to metabolic stress in AD.^(68)^ Among the enzymes affected in this process are NAD(P)H oxidase (NOx), cytochrome p450, monoamine oxidase enzyme (MAO), xanthine oxidase (XO), nitric oxide synthase (NOS), and myeloperoxidase (MPO).^(69)^

Studies carried out from brain tissue biopsies of AD patients have shown the presence of mitochondrial DNA in the cell cytoplasm, as well as 8-hydroxyguanosine markers and nitrotyrosine, which indicate mitochondrial destruction during the progression of the disease. The accumulation of metals, such as copper (Cu) and zinc (Zn), also contributes to synaptic dysfunctions caused by oxidative lesions. Under normal conditions, both act in synaptic modulation, and Cu blocks NMDA receptors so that calcium entry into the cell is limited. If there is an imbalance in homeostasis of the transition metals, they can bind to oxygen and generate toxic products in the central nervous system.^(70)^

Some environmental factors can also aggravate oxidative damage, such as ultraviolet radiation, lifestyle, alcoholism, smoking, and specific pollutants.^(71,72)^ In general, oxidative stress can suppress the antioxidant activity of human cells, impair calcium homeostasis, generate protein accumulation, alter functions of the proteasome, damage the cell membrane, and cause more mitochondrial dysfunction, resulting in a vicious circle of oxidative damage.^(73,74)^

### ApoE4 hypothesis

Genetic factors related to AD are used to understand individual risk profiles. These factors are considered a bridge between the clinical picture and basic research. Among these factors, apolipoprotein E (ApoE) is related to pathogenic mechanisms of AD, and of great importance for better understanding of neurodegenerative disease and even for possible new therapeutic targets.^(75)^

Apolipoprotein E is a polymorphic lipoprotein with three isoforms in common: ApoE2, ApoE3, and ApoE4, in humans. These lipoproteins are produced mainly by astrocytes and play an essential role in transporting cholesterol through ApoE receptors. The three isoforms differ from each other by replacing one or two amino acids at positions 112 and 158, as follows: ApoE2 has a cysteine residue at both positions; ApoE3 has an arginine at position 158; while type 4 has an arginine residue at both positions.^(76)^ The polymorphisms presented by the ApoE protein are the primary genetic risk factor for AD development. Homozygous individuals for the ApoE4 alleles have a greater than 50% risk for the AD phenotype, while for a heterozygous individual, the risk reduces to something between 20% and 30%.^(77)^ The most common mechanism by which ApoE4 participates in AD development is increasing the aggregation of beta-amyloid plaques and reducing their clearance. Other mechanisms associated with ApoE4 are related to tauopathy, neuroinflammation, and reduction in the rate of glucose metabolism in the parietal and temporal region of the brain in AD patients. In response to neuroinflammation, ApoE4 undergoes neuron-specific proteolysis, which results in fragments of toxic bioactive substances responsible for interfering with the mitochondrial energy balance.^(30)^

Apolipoprotein E4 in the pathogenesis of AD related to beta-amyloid plaques: *in vivo*, ApoE is associated with amyloid plaques, and *in vitro*, free ApoE3 and ApoE4 can form stable complexes with beta-amyloid plaques with ApoE4 binding more quickly and firmly than ApoE3.^(78)^ There are reports of mechanisms in ApoE-deficient rats expressing the humanized amyloid precursor protein (hAPP) through a mutation, showing that ApoE is necessary to form fibrillar amyloid plaques. Even so, ApoE also contributes to clearance of beta-amyloid, where ApoE2 and ApoE3 contribute more to this task than ApoE4.^(79)^ A study measured the beta-amyloid clearance rate by microdialysis in hAPP transgenic mice, expressing ApoE3 and ApoE4, and showed ApoE4 decreases beta-amyloid clearance by 40% when compared to ApoE3.^(80)^ *In vivo* and *in vitro* studies suggested the low-density lipoprotein (LDL) receptor and the very low-density lipoprotein (VLDL) receptor are involved in the beta-amyloid clearance of the ApoE-dependent.^(81)^ Thus, ApoE4 is related to the decrease in beta-amyloid clearance, causing accumulation and the consequent formation of beta-amyloid plaques in the brain of humans and models of transgenic mice.

### GSK3 hypothesis

Glycogen synthase kinase 3 is a type of serine/threonine kinase enzyme by constitutive and ubiquitous proline involved in various cellular processes, which has two isoforms, α, and β, and are closely related to hyperphosphorylation of tau protein, impaired memory, and increased accumulation of beta-amyloid plaques.^(82)^ Glycogen synthase kinase 3 beta (GSK3β) has also been shown to reduce the synthesis of acetylcholine, which agrees with the cholinergic deficit presented in AD.^(83)^ Furthermore, GSK3β is a critical mediator in cell apoptosis, thus contributing to neuronal loss seen in AD.^(84)^

Glycogen synthase kinase 3 proved to be a beneficial biomarker for AD diagnosis through the GSK3β assay.^(85)^ In addition, several studies have already reported an interaction between GSK3 and oxidative stress in neuroinflammation.^(86)^ Moreover, GSK3 potentiates the process of apoptosis and cell death by regulating the transcription of various factors, such as heat shock factor-1 (HSF-1), cyclic-AMP response element-binding protein (CREB), and nuclear factor Kappa B (NF-κB). These transcription factors play a vital role in neurodegeneration.^(30)^ As GSK3 has a central activity in the pathogenesis of AD, it would be valid to say that its enzyme activity is increased in patients affected by the disease. However, there is little evidence pointing to this fact, since it is technically difficult, if not impossible, to measure the enzymatic activity in *post-mortem* brain tissues.^(82)^ Nonetheless, the association of GSK3 with other pathogenesis pathways of AD is a subject to be explored. Once the relation between GSK3 and beta-amyloid generation be clear, GSK3 inhibitors may be a new therapeutic target for AD.^(30)^

## CREB

The responsive element to cyclic adenosine monophosphate (cAMP) is a cellular transcription factor involved in regulating several downstream genes, such as somatostatin, encephalin, corticoliberin, and a circadian period protein homolog.^(30)^ Cyclic AMP response element-binding protein is vastly produced in the brain, particularly in the regions essential for memory coding and learning, including hippocampus and cortex. Its phosphorylation occurs in a serine residue at position 133 through various protein kinases, such as calcium/calmodulin-dependent protein kinase (CaMKII and CaMKIV), MAPK and PKA.^(87)^ Some evidence shows the CREB signaling pathway mediates the effect of beta-amyloid on hippocampal synaptic plasticity and memory loss. The first study demonstrating this relation was carried out by Vitolo et al., where beta-amyloid 42 decreased PKA activity and interrupted long-term potentiation in hippocampal slices.^(88)^ The *deficit* in neuronal plasticity induced by beta-amyloid is associated with reduced levels of CREB phosphorylation by a mechanism that may involve the endocytosis of NMDA through calcineurin.^(89)^ Low levels of cAMP and changes in the composition of the PKA regulatory or catalytic subunits caused by the accumulation of beta-amyloid are associated with a decrease in the CREB phosphorylation rate in neuronal cultures and brains with AD.^(88)^ Cyclic AMP response element-binding protein participates in the coding of memory and extinction of some types of memories. Since the molecular mechanism by which CREB increases synaptic efficacy and memory processing involves the regulation of gene transcription, a plausible hypothesis to understand memory in a pathological condition may include the activation of specific genes linked to memory.^(87)^ Specific CREB-dependent genes that regulate neuronal functions have already begun to emerge,^(90)^ while a general mismatch of CREB target genes appears to occur in the hippocampus of patients with dementia.^(91)^ Understanding this network of gene expression can provide new approaches to unfold new therapeutic targets for AD.

### Alzheimer’s disease as type 3 diabetes

About 80% of people with AD have insulin resistance or type 2 diabetes. Considerable evidence indicates that some metabolic syndromes contribute to the development and worsening of AD.^(92)^ Insulin signaling in the brain plays a critical role in regulating food intake, body weight, reproduction, learning, and memory.^(93)^ It has already been shown that intranasal administration of insulin improves working memory in humans and animals.^(94)^ In addition, intrahippocampal insulin administration strengthens hippocampus-dependent spatial memory.^(95)^ Furthermore, the levels of messenger RNA encoding insulin receptors and protein levels of receptors are increased in the formation of short-term memory after a spatial memory task, suggesting that neuronal sensitivity to insulin may be increased during learning.^(96)^

The condition of insulin resistance can lead to neuronal impairment and loss of cognitive abilities, accompanied by increased insulinemia and relative inactivity in peripheral regions. Consequently, this leads to the development of neuritic plaques, hippocampal atrophy, cognitive *deficit*, and decreased cerebrocortical glucose metabolism, which is closely correlated with memory *deficits* presented in AD.^(97)^ Due to lack of insulin response, negative regulation of insulin receptors, reduced binding of insulin to receptors, or even the defective activation of insulin signaling cascade, the main consequences generated are decreased uptake of neuronal glucose that manifests with impaired neuroplasticity, *deficits* in neurotransmitters, collapse of energy mechanism, and beginning of the cascade of destructive inflammatory processes. In general, these consequences may explain the relation between obesity, type 2 diabetes, and AD.^(97)^

Due to the increased regulation of advanced glycation end products, they have also been associated with increased deposition of beta-amyloid plaques, which, in turn, promotes the formation of neurofibrillary tangles by increasing the expression of neuroinflammatory mediators, such as IL-6 and TNF-α.^(30)^ In addition, studies showed insulin signaling regulates various stages of the amyloid cascade and affects beta-amyloid aggregation in the brain. Insulin increases the transcription of anti-amyloidogenic proteins, for instance, the insulin-degrading enzyme and α-secretase, and stimulates beta-amyloid clearance.^(92)^

However, the connections between insulin resistance and AD do not yet provide enough details to advance in developing new molecules that increase sensitivity of receptors to insulin. Several new mice models need to be used to allow gene editing specifically for insulin signaling in the brain.^(30)^

### Alzheimer’s disease and herpes simplex virus

The herpes simplex virus type 1 has a marked tropism for the nervous system and remains latent in the trigeminal ganglion of the peripheral nervous system in the infected individual.^(21)^ In 1982, Ball suggested that during the reactivation of the microorganism, it can ascend through nerve pathways that reach the limbic system and cause acute infections by generation of virions, so that they reach areas related to AD, such as hippocampus, frontal and temporal lobes.^(98)^

The herpetic infection triggers several metabolic imbalances that could contribute to the development of cognitive dementia. As soon as it emerges from the latent form, the herpes virus can cause tau protein hyperphosphorylation by suppressing the enzymes GSK3β and PKA.^(99)^ Furthermore, it affects apoptosis, inducing or blocking it through viral proteins,^(100)^ and impairs the autophagy of neuronal cells, which leads to accumulation of autophagosomes and increased intracellular levels of beta-amyloid peptide.^(101)^ The latter change can also be caused by homeostatic calcium dysregulation induced by the herpetic infection.^(102)^

Herpes simplex virus type 1 also causes mitochondrial dysfunctions and damage and induces oxidative stress by producing reactive oxygen species that surpass the antioxidant barrier of cellular defense, and generate an inflammatory response characterized by increased pro-inflammatory cytokines that determine neuroinflammation. Finally, the occurrence of synaptic dysfunctions has also been reported. It appears to be influenced by several factors, such as accumulation of beta-amyloid and hyperphosphorylation of the tau protein, in addition to reduction in CREB activity and other proteins that participate in synaptic transmission.^(102)^ The ApoE4 allele associated with herpes simplex infection can significantly increase the risk of developing AD.^(103)^

*In vitro* studies have shown that animal cells infected by herpes simplex virus type 1 showed an increase in pro-inflammatory mediators for AD, such as IL-1b, IL-6, and IFN-g.^(104)^

### Endocannabinoid system and Alzheimer’s disease

Cannabinoids are natural compounds found in *Cannabis sativa*. The plant contains more than 500 compounds, and at least 85 of them are named cannabinoids. The best known of these is delta-9-tetrahydrocannabinol. Delta-9-tetrahydrocannabinol elicits psychoactive effects when it binds to the cannabinoid receptors on the cell membrane.^(105)^ These receptors are present both in the central nervous system and peripheral regions of the human body and are classified as type 1 (CB1) and type 2 (CB2). The signaling pathway by which CB1 and CB2 act has participation in a variety of physiological functions and several pathophysiological functions.^(106)^ The discovery of the endogenous compounds anandamide, also known as N-arachidonoylethanolamine, and 2-arachidonoylglycerol, which activate cannabinoid receptors, emphasized the importance of the endocannabinoid system in regulating a variety of biological functions and its relation with neurodegenerative diseases.^(107)^

The distribution of CB1 receptors in adult rodent brains is highly heterogeneous, with the highest density in the basal ganglia, including the substantia nigra and hippocampus, particularly in the dentate gyrus and the molecular layer of the cerebellum.^(108)^ This distribution is similar in the human brain. The cognitive *deficits* presented by AD patients correlate with a cerebral disorder in sensitive areas of the brain, mainly in the frontal cortex and the hippocampus, which are rich in CB1.^(109)^ Correlation analyses based on histological sections of *post*–*mortem* brain tissues from patients with confirmed AD indicated low levels of CB1 when compared to control groups with corresponding age.^(110)^

Significant changes in the molecular architecture, cell identity, and function of 2- arachidonoylglycerol metabolism have been described in histological sections of patients with confirmed AD, as well as in transgenic mice or pharmacological models of AD.^(111)^

Cannabinoid receptor agonists, such as anandamide and noladin, have been shown to protect against neurotoxicity caused by beta-amyloid in differentiated neuronal cells through binding to the CB1 receptor and activation of the MAPK pathway.^(112)^ The increased concentration of 2- arachidonoylglycerol through the pharmacological inhibition of monoacylglycerol lipase robustly suppressed the production and accumulation of beta-amyloid plaques associated with decreased expression of the enzyme that participates in the synthesis of beta-amyloid in model mice with AD. Monoacylglycerol lipase inhibition also reduced neuroinflammation, decreased neurodegeneration, maintained the function integrity and synaptic structure in the hippocampus, and improved long-term synaptic plasticity and learning, as well as spatial memory in animals with AD.^(113)^

The non-psychoactive phytocannabinoid, cannabidiol has been shown to be neuroprotective *in vitro* and *in vivo*.^(114)^ Neuronal cells pre-treated with cannabidiol showed decreased expression of the NOS enzyme and reduced hyperphosphorylation of the tau protein after treatment with beta-amyloid.^(115)^ In addition, the levels of reactive oxygen species, products of lipid peroxidation, and activation of caspase-3 were also attenuated by cannabidiol.^(116)^ These results show the relation between the endocannabinoid system with oxidative stress and neuroinflammation, which are strong characteristics of AD, bring new molecular knowledge about the neuroprotective effect of cannabidiol, and suggest a possible role in the pharmacological management of AD, especially given its low toxicity in humans.^(115)^

## DISCUSSION

**Figure 1:**
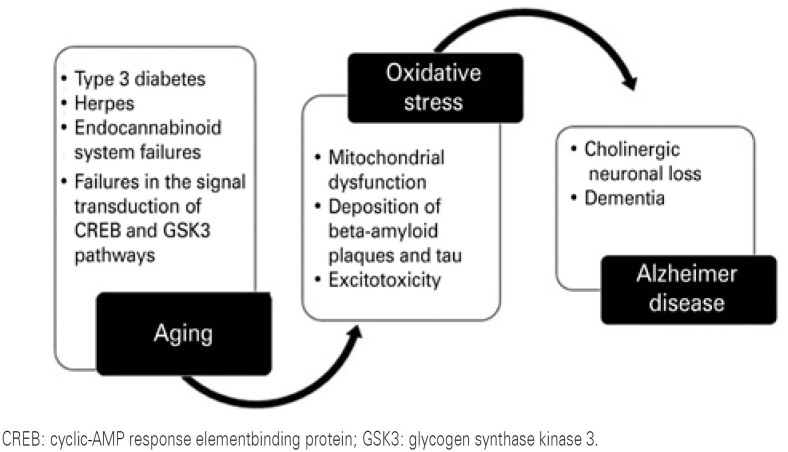
Possible ontogenetic scheme of Alzheimer’s disease pathophysiology

By describing all these mechanisms related to pathophysiology of AD, an association among them could be made, leading to causes that generate the clinical picture of patients. With aging, there is a progressive accumulation of oxidative stress and ApoE4 due to excitotoxicity, failures in the endocannabinoid system, type 3 diabetes, or even herpes simplex virus. These factors cause an imbalance in mitochondrial function and various signal transduction cascades, such as the CREB and GSK3 pathways. All these processes lead to dysfunctions in the synthesis, metabolism, and degradation of macromolecules that already have a well-described relation with the pathophysiology of AD, including beta-amyloid plaques and tau proteins. The neurons that are more sensitive for being at the crossroads of nervous circuits, such as the hippocampal and cholinergic neurons, are responsible for maintaining optimal functioning of these regions, and are the first to be affected by the sum of all these dysfunctions. However, the cholinergic neuronal loss, which is the target of all the therapeutics available so far, is only the final consequence of all processes, and far from being the cause (Figure 1).

With the natural aging of an individual, there is an accumulation of oxidative stress due to excitotoxicity, which may be due to failures in the endocannabinoid system, type 3 diabetes, or even herpes. All these processes can cause mitochondrial dysfunction and interfering in signal transduction pathways of transcription and protein translation. Due to these dysfunctions, the metabolism, synthesis, and degradation of some macromolecules, beta-amyloid and tau, are impaired, occurring in neuronal regions. The most sensitive neurons are the first to be affected by the sum of all these dysfunctions since they are located at the crossroads of the nervous circuit, such as the cholinergic ones, which are responsible for the optimal functioning of these regions. Thus, cholinergic neuronal loss is only a final consequence of the disease, and far from being the cause.

Another factor to be considered is the high energy demand that neurons have to maintain their functioning. Many of them have their glycolytic capacity diminished and thus are highly dependent on the energy generated in mitochondria. Besides the role in the energy supply, mitochondria also play a role in the metabolism of macromolecules and steroids, production of free radicals, intracellular control of calcium levels, and apoptosis,^(117)^ all mechanisms involved in neurodegeneration. Given the significant role of mitochondria in controlling concentration of reactive oxygen species (ROS), both acting as an antioxidant agent and via production, since it is the organelle specialized in oxidation, with several enzymes involved in the superoxide production. Therefore, damage to mitochondria can result in increased oxidative stress via ROS accumulation, which can lead to damage to DNA, RNA, proteins, and lipids.^(118)^

Furthermore, several *in vitro* studies have suggested a bridge between elevated beta-amyloid levels with mitochondrial dysfunction and oxidative stress, which are closely linked to pathophysiology of AD.^(119)^ Moreover, it has already been reported that beta-amyloid is a key factor in the free radical generation oxidative damage, activating a cascade of events that lead to neurodegeneration in neuroblastoma LAN5 cells.^(120)^ Based on these pieces of information, it is easy to conclude that mitochondrial dysfunction is essential in pathophysiology of AD and its worsening over time. The endocannabinoid system has significant importance in this issue, for it regulates antioxidant activities and plays a vital role in reducing the deposition of beta-amyloid plaques. The failure of this system may be related to neuroinflammation and accumulation of oxidative stress. These relations may direct new research targeting the endocannabinoid system for the possible generation of new drugs to treat and prevent AD.

Recently a new drug was approved for treatment of AD and it that does not target the cholinergic system. It is donanemab, a humanized IgG1 antibody directed to an epitope on pyroglutamate at the N-terminus of beta-amyloid, which is present only in established beta-amyloid plaques and is specific for this type of beta-amyloid plaque, showing no binding to other beta-amyloid species. In patients with early AD, donanemab showed better scores on cognition tests and ability to perform daily tasks than placebo at 76 weeks, although secondary endpoints were mixed.^(121)^ More extensive and more prolonged trials are warranted to study the efficacy and safety of donanemab in AD. However, there is hope for this treatment, since it is the first drug with a different therapeutic target for those who already used some based on the cholinergic system. In this way, the disease can be combated earlier, thus avoiding the final consequences of the pathophysiological cascade of AD, *i.e*., cholinergic neuronal loss.

## CONCLUSION AND FUTURE PROSPECTS

This review addressed the physiological mechanisms from the most accepted or most described in the scientific community, the amyloid hypothesis and tau, as well as some emerging mechanisms related to the pathophysiology of Alzheimer’s disease. All mechanisms described have some relation between them that can be described and generate a better understanding to achieve new therapeutic targets for Alzheimer’s disease.

The cause of Alzheimer’s disease is still far from being described. Many mechanisms point to the clinical picture presented by patients. A relation among all mechanisms described or failure of some systems or pathways seem to be the pathophysiological pathway of the disease. However, further studies are warranted to understand the pathophysiology of the disease and to advance future translational research with new therapeutic targets, acting to reduce cholinergic neuronal loss or even halt it.

The endocannabinoid system is of foremost importance for many processes that are deregulated and could be an important therapeutic target to reduce oxidative stress. Nevertheless, the relation of the receptors of this system with memory has not been very well described. In the future, we will seek to discover this relation through animal models evaluating their spatial memory. Afterwards, it will be possible to reveal if agonist or antagonist response of receptors has some beneficial or harmful effect on reconsolidation of spatial memory, and which pathways are active and participate in this process. Preclinical research is of the utmost importance to unravel this pathophysiological process presented in Alzheimer’s disease, and that is our future aim.
